# Mitochondrial genome of *Dinophilus gyrociliatus* (Annelida: Dinophilidae)

**DOI:** 10.1080/23802359.2017.1407704

**Published:** 2017-11-25

**Authors:** Kyle T. David, Kenneth M. Halanych

**Affiliations:** Molette Biology Laboratory for Environmental and Climate Change Studies, Department of Biological Sciences, Auburn University Auburn, AL, USA

**Keywords:** Mitochondrial genome, annelid, gene-order

## Abstract

Here we report the 14,678 bp mitochondrial genome of the annelid *Dinophilus gyrociliatus*, the first mitochondrial genome from Dinophilidae. We recovered 13 protein-coding genes, two rRNA, and 21 tRNA, the order of which is different from other annelid species. Interestingly, trnS1 was not recovered. The GC% across the genome was 34.20%.

*Dinophilus gyrociliatus* is a small interstitial polychaete worm which inhabits littoral zones worldwide (Prevedelli and Vandini 1999; Prevedelli and Simonini 2000). They undergo dimorphic programmatic sex determination; males are ∼50μm long whereas females may reach 1.2 mm (Windoffer and Westheide 1988). Males possess no organs save those required for reproduction and die shortly after fertilizing their sisters (Åkesson and Costlow 1991). These features make *D. gyrociliatus* a popular candidate for studying sex ratio and determination (Åkesson and Costlow 1991; Prevedelli and Vandini 1999; Prevedelli and Simonini 2000; Simonini and Prevedelli 2003). Relatively easy to culture, *D. gyrociliatus* also make a good model for neurological studies (Müller and Westheide 2002; Fofanova and Voronezhskaya 2012). Although Dinophilidae were once placed close to dorvilleid annelids, their phylogenetic affinities are uncertain (Figure 1) (Struck et al. 2015).

**Figure 1.**
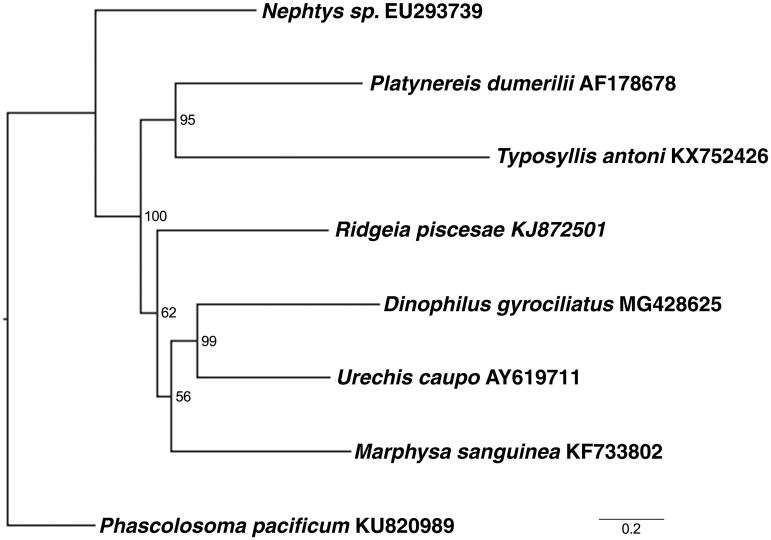
Maximum-likelihood tree with boostrap support (1000 iterations) of concatenated nucleotide sequenes for thirteen mitochondrial protein-coding genes in seven Pleistoannelid species (see text). The GTRGAMMA model was employed using RAxML (Stamatakis 2014). *Phascolosoma pacificum* – KU820989.1 was used as the outgroup.

Animals were originally collected from fouling material on dock pilings near the Duke Marine Laboratory, Beaufort, NC (34°43′04″N 76°40'14″W). Cultures were obtained from the late Bertil Åkesson in 2000 and grown on a spinach diet. Multiple individuals were harvested and frozen at −80 °C until their DNA was extracted in 2012 using a Qiagen DNEasy blood and tissue extraction kit (Qiagen Inc., Valencia, CA) according to the protocol from the manufacturer. Total genomic DNA was prepared with Illunina’s Nextera DNA sample preparation kit (Illumina, San Diego, CA) and run on an Illumina MiSeq sequencer using a 2 × 250 paired-end protocol in the Molette Laboratory, Department of Biological Sciences, Auburn University. Mitochondrial genomes were assembled de novo using Ray 2.2.0 (Boisvert et al. 2010) after digital normalization. To identify putative mitochondrial contigs, BLASTn (Altschul et al. 1997) was employed with the *Riftia pachyptila* mitochondrial genome (GenBank Accession AY741662; Jennings and Halanych 2004) as a bait. One contig was recovered which was long enough to represent the entire mtDNA genome. This contig was annotated using MITOS 2 (Bernt et al. 2013) and gene boundaries were compared manually with published annelid mitochondrial genomes.

The mitochondrial genome of *Dinophilus gyrociliatus* (GenBank Accession MG428625) is 14,678 bp long, making them the eighth shortest (12th percentile) of the 70 annelid mitochondrial genomes currently listed on Genbank. The overall nucleotide composition is as follows: A = 33.9% (4978 bp), C = 11.8% (1738 bp), G = 22.3% (3283 bp), and T = 31.8% (4679 bp). A GC content of 34.20% puts the *D. gyrociliatus* mitochondrial genome in the 47th percentile among annelids.

Thirteen protein-coding genes were found, consistent with other animal mitochondrial genomes. The ribosomal RNA rrnL was not initially recovered by MITOS (Bernt et al. 2013) but it was subsequently identified via a Blastn search of positions 4369–5457. Surprisingly trnS1, a transfer RNA prevalent throughout animal mitochondrial genomes, was absent from searches in both MITOS and ARWEN v1.2 server (Laslett and Canbäck 2007).

Mitochondrial gene order is expected to be conserved in Pleistoannelida, of which *Dinophilus* is a member (Struck et al. 2015). However, the ATP6 to NAD5 block was found switched with the adjacent COX3-NAD6-CYTB block in *D. gyrociliatus* compared with the hypothesized ground state of mtDNA for Pleistoannelida (Weigert et al. 2016). As anticipated, tRNA gene order is less conserved, no two tRNAs remain consistently adjacent across seven Pleistoannelids (including *Marphysa sanguinea* – KF733802.1, *Nephtys* sp – EU293739.1, *Platynereis dumerilii* – AF178678.1, *Typosyllis antoni* – KX752426.1, *Ridgeia piscesae* – KJ872501.1, and *Urechis caupo* – AY619711.1).

## References

[CIT0001] Åkesson B, Costlow JD. 1991 Effects of constant and cyclic temperatures at different salinity levels on survival and reproduction in *Dinophilus gyrociliatus* (Polychaeta: Dinophilidae). Bull Marine Sci. 48:485–499.

[CIT0002] Altschul SF, Madden TL, Schäffer AA, Zhang J, Zhang Z, Miller W, Lipman DJ. 1997 Gapped BLAST and PSI-BLAST: a new generation of protein database search programs. Nucleic Acids Res. 25:3389–3402. 9254694 10.1093/nar/25.17.3389 PMC146917

[CIT0003] Bernt M, Donath A, Jühling F, Externbrink F, Florentz C, Fritzsch G, Pütz J, Middendorf M, Stadler PF. 2013 MITOS: improved de novo metazoan mitochondrial genome annotation. Mol Phylogenet Evol. 69:313–319. 22982435 10.1016/j.ympev.2012.08.023

[CIT0004] Boisvert S, Laviolette F, Corbeil J. 2010 Ray: simultaneous assembly of reads from a mix of high-throughput sequencing technologies. J Comput Biol. 17:1519–1533. 20958248 10.1089/cmb.2009.0238 PMC3119603

[CIT0005] Fofanova E, Voronezhskaya E. 2012 The structure of archiannelid *Dinophilus gyrociliatus* ventral nerve cords. Acta Biol Hungar. 63:88–90. 10.1556/ABiol.63.2012.Suppl.2.11 22776479

[CIT0006] Jennings RM, Halanych KM. 2004 Mitochondrial genomes of *Clymenella torquata* (Maldanidae) and *Riftia pachyptila* (Siboglinidae): evidence for conserved gene order in Annelida. Mol Biol Evol. 22:210–222. 15483328 10.1093/molbev/msi008

[CIT0007] Laslett D, Canbäck B. 2007 ARWEN: a program to detect tRNA genes in metazoan mitochondrial nucleotide sequences. Bioinformatics. 24:172–175. 18033792 10.1093/bioinformatics/btm573

[CIT0008] Müller M, Westheide W. 2002 Comparative analysis of the nervous systems in presumptive progenetic dinophilid and dorvilleid polychaetes (Annelida) by immunohistochemistry and cLSM. Acta Zool. 83:33–48.

[CIT0009] Prevedelli D, Simonini R. 2000 Effects of salinity and two food regimes on survival, fecundity and sex ratio in two groups of *Dinophilus gyrociliatus* (Polychaeta: Dinophilidae). Marine Biol. 137:23–29.

[CIT0010] Prevedelli D, Vandini RZ. 1999 Survival, fecundity and sex ratio of *Dinophilus gyrociliatus* (Polychaeta: Dinophilidae) under different dietary conditions. Marine Biol. 133:231–236.

[CIT0011] Simonini R, Prevedelli D. 2003 Effects of temperature on two Mediterranean populations of *Dinophilus gyrociliatus* (Polychaeta: Dinophilidae): I. Effects on life history and sex ratio. J Exp Marine Biol Ecol. 291:79–93.

[CIT0012] Stamatakis A. 2014 RAxML version 8: a tool for phylogenetic analysis and post-analysis of large phylogenies. Bioinformatics. 30:1312–1313. 24451623 10.1093/bioinformatics/btu033 PMC3998144

[CIT0013] Struck TH, Golombek A, Weigert A, Franke FA, Westheide W, Purschke G, Bleidorn C, Halanych KM. 2015 The evolution of annelids reveals two adaptive routes to the interstitial realm. Curr Biol. 25:1993–1999. 26212885 10.1016/j.cub.2015.06.007

[CIT0014] Weigert A, Golombek A, Gerth M, Schwarz F, Struck TH, Bleidorn C. 2016 Evolution of mitochondrial gene order in Annelida. Mol Phylogenet Evol. 94:196–206. 26299879 10.1016/j.ympev.2015.08.008

[CIT0015] Windoffer R, Westheide W. 1988 The nervous system of the male *Dinophilus gyrociliatus* (Annelida: Polychaeta). I. Number, types and distribution pattern of sensory cells. Acta Zool. 69:55–64.

